# Advantages and Pleasures of a Physician’s Life

**Published:** 1854-02

**Authors:** 


### *Advantages and Pleasures of a Physician's Life.*

FROM THE LATE INTRODUCTORY LECTURE OF PROF. JONATHAN KNIGHT, OF THE MEDICAL INSTITUTION OF YALE COLLEGE.

Another advantage of the medical profession is, that the mind of the physician is not, and if he is in any degree faithful to his duties cannot be, continually occupied with mere pecuniary matters. He has a right, to be sure, to look forward to a fair remuneration for his services, and usually receives it; but his mind must be mainly occupied with other and higher interests. His duty to his patients, his anxiety for their recovery, his careful study of their diseases and of the means of relieving them, will engross his best and most diligent thoughts, and he will soon find that there are other books more interesting than his day book and ledger. It is a misfortune attached to any employment, that its pecuniary results are its principal attraction. The business that is necessarily begun and pursued under the influence of the often repeated and much praised maxim, a penny saved is two pence clear, and a pin a-day is a groat a-year, cannot do much in elevating the mind, or ennobling the feelings, or in raising the man much above the earth on which he dwells.

It is true that such employments may be and often are followed by the liberal minded man, without self deterioration; still their tendency is to belittle the mind, and to narrow the feelings into the compass of selfishness. I do not mean by this that a business is to be avoided as injurious merely because it is profitable; a gainful employment may be safely followed, so long as the occupation which it gives to the body and mind is its principal attraction.

The mechanic may construct a machine in the hope of a large erward, and yet receive a far higher gratification from the successful exertion of his faculties, and this may be and is often the controlling motive of his labor rather than the pecuniary result. The merchant while his gains are counted by thousands, yet may be more richly rewarded by the consciousness that he is the controlling mind of large and important interests, that he is successful in developing the resources of hiscountry, and that he is ministering largely to the happiness of his fellow men. In all suchcases though accumulation may follow thrift, yet to accumulate is not made the chief purpose of life. From the temptation so prevalent in many other employments, to pursue gain with greedinesss, the physician is guarded, by the full and constant pre-occupation of his mind by other and higher thoughts, as well as by the early learnt truth that such a pursuit will be unavailing. The physician who engages in his business with the purpose of becoming rich uppermostin his heart, is very apt soon to leave it, and to become the nostrum monger, or the "pathy" follower, or to engage in some pursuit more congenial to his spirit, and more likely to gratify his desire. This freedom from temptation to petty gains, and resulting avarice growing out of the business of the physician, is one of its important advantages.

Another advantage of the profession, and which contributes largely to the happiness of the physician, is that it compels him to possess or to assume cheerfulness of disposition, kindness of demeanor, and a readiness to perfom acts of beneficence. These constitute no inconsiderable portion of his stock in trade, and without a liberal share of them he will soon become a bankrupt. He must be kind to his patients, considerate of their felings, patient of their complaints though they may often seem to be unreasonable, and ready to afford them consolation and relief. He must therefore cultivate these feelings until they become a part of his very constitution. He who commences a course of this kind from the necessity of his position, will soon learn to continue it from the love it; as the features which often called upon to express any one of the strong emotions of the mind, joy or sorrow, pleasure or pain, will become ultimately formed or moulded, so that what at first was transient, will become their habitual expression, so the mind into which any strong emotions habitually enter, whether of necessity, and more especially, when of choice, falls more and more under the influence of such feelings, until it become the controlling power of its actions. This in-working of the kind feelings which he is so often called upon to express and to experience, is so effective, that it is rare to find a physician advanced in life, who is other than a cheerful, social and benevolent man. The influence of this state upon his own happiness can hardly be over-estimated. It is a law of our nature as definite and as operative as the law of gravitation in its effects upon material bodies, that to do good to others, is to gain it for ourselves, and that our own happiness is very nearly in proportion to the active exertions which are made to promote the happiness of our fellow men.

After all, however, the chief source of the physician's enjoyment springs from the successful result of his efforts to relieve distress, remove disease, and rescue the dying from their danger. As very much of his often disheartening anxiety and despondency is occasioned by the unfavorable termination of the cases submitted to his care, so his greatest joy grows out of those which end favorably. And it is to be remembered that by far the larger number of cases, even of of dangerous diseases, end in recovery. There is a pleasure unappreciable except by experience, in the consciousness of power over disease and of ability to conduct it to a favorable end; there is joy that the life of one dear to many and perhaps important to the community, is saved; there is the feeling of gratitude fairly earned and often liberally bestowed. It is doubtful whether the physician ever feels a more honest and gratifying elation, than upon the recovery of his first patient from dangerous sickness. It is difficult to describe the emotion of the physician, who after long watching the progress of a dangerous sickness, first sees the light of hope breaking in upon the darkness with which it has been shrouded.

